# A biphenotypic lymphocyte subset displays both T- and B-cell functionalities

**DOI:** 10.1038/s42003-023-05719-9

**Published:** 2024-01-05

**Authors:** Yifan zhang, Cuiyuan Guo, Yigong Zhou, Wenhong Zhang, Zhaoqin Zhu, Wanhai Wang, Yanmin Wan

**Affiliations:** 1 Clinical Laboratory, The First Affiliated Hospital of Zhengzhou University, Key Laboratory of Laboratory Medicine of Henan Province, Zhengzhou, China; 2 Department of Infectious Diseases, Shanghai Key Laboratory of Infectious Diseases and Biosafety Emergency Response, National Medical Center for Infectious Diseases, Huashan Hospital, Shanghai Medical College, Fudan University, Shanghai, China; 3 Department of Laboratory Medicine, Shanghai Public Health Clinical Center, Shanghai, China; 4 Life Science Department, Faculty of Agricultural and Environmental Sciences, Macdonald Campus of McGill University, Quebec, Canada; 5 Shanghai Huashen Institute of Microbes and Infections, Shanghai, China

**Keywords:** Lymphocytes, Physiology

## Abstract

T cell/B cell mixed phenotypic lymphocytes have been observed in different disease contexts, yet their presence and function in physiological conditions remain elusive. Here, we provide evidence for the existence of a lymphocyte subset endogenously expressing both T- and B-cell lineage markers in mice. The majority of these T/B phenotypic lymphocytes (CD3^+^CD19^+^) show an origin of pro/pre B cells and distribute widely in mouse bone marrow, lymph nodes, spleen, and peripheral blood. Functional assays show that these biphenotypic lymphocytes can be activated through stimulating TCR or BCR signaling pathways. Moreover, we show that these cells actively participate both the humoral and cellular immune responses elicited by vaccination. Compared to conventional T cells, these biphenotypic lymphocytes can secrete a higher level of IL-2 but a lower level of TNF-α upon antigen specific stimulation. An equivalent lymphocyte subset is found in freshly isolated human PBMCs and exhibits similar functionality, albeit at a lower frequency than in mice.

## Abstract (web summary)

This study identifies T/B phenotypic lymphocytes distributed across various mouse organs, potentially originating from pro/pre B cells, and demonstrating functional activity in immune responses.

## Introduction

The development of conventional T and B lymphocytes is fine and strictly tuned^1^. The phenotypical differences between T and B cells are usually thought to be clear, which can be readily discerned through detecting cell surface markers such as CD19, CD20, CD3, CD4, CD8, BCR, and TCR. However, accumulating evidence indicates that the boundary between T and B cells could be ambiguous under both pathological and physiological circumstances.

T/B biphenotypic lymphocytes that co-express T- and B-cell lineage markers are well documented in hematopoietic malignancies. Aberrant presence of B-cell-associated antigens such as CD20 and CD79a was observed in T-cell lymphomas^2,3^. Meanwhile, T-cell-associated antigens such as CD3, CD4, CD8, and CD5 were also detected in B-cell lymphomas^4,5^ and plasma cell tumors^6,7^. CD3^+^ T cells carrying B-cell-associated marker (CD20) had also been observed in both healthy individuals and patients with immune dysregulation^8–15^. These biphenotypic cells are believed to play functional roles in multiple diseases, such as multiple sclerosis^14^, HIV infection^16^, rheumatoid arthritis^11^ and ovarian cancer^10^. Despite of being intensively observed, the origin of these biphenotypic lymphocytes is controversial. There is evidence that shows CD3^+^CD20^+^ cells are unable to endogenously express CD20, and instead acquire CD20 from B cells via trogocytosis^10,14,17^. Conversely, other evidence suggests that CD3^+^CD20^+^ T cells represent a unique subset of T cells which endogenously expresses CD20^15,18–20^.

Different from the abovementioned studies, recent studies reported a previously unknown lymphocyte subset that co-expressed CD3 and CD19 in human^21,22^. This subset of lymphocytes might play crucial roles in type I diabetes^21^ and infectious diseases^22^. However, its presence and function in normal physiological conditions have not been fully demonstrated. In this study, we showed that the CD3^+^CD19^+^ lymphocyte subset could be detected in both healthy mice and humans. More intriguingly, our data proved that this lymphocyte subset could carry out the typical functions of both conventional T and B cells. Our study offered a fresh perspective on the complexity of the immune system.

## Results

### Single cell RNA-seq unveiled a subset of T/B biphenotypic lymphocytes in mice

**Fig. 1:**
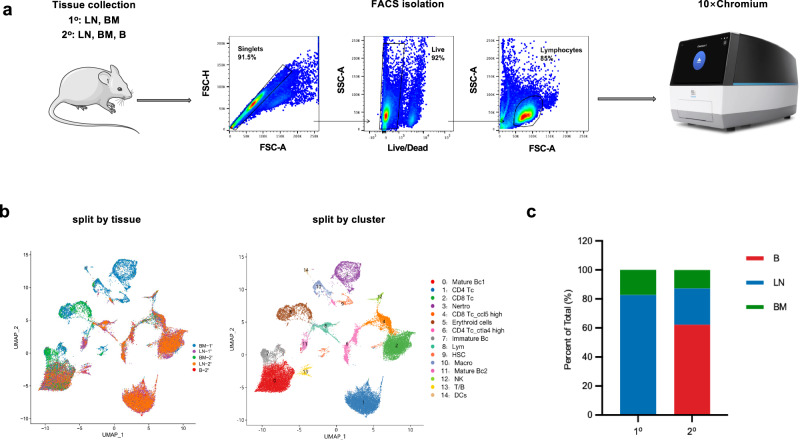
Single cell RNA-seq unveiled a subset of T/B biphenotypic lymphocytes in mice. **a** Lymphocytes isolated from mouse BM (bone marrow), LN (lymph nodes) and B (blood) were firstly purified using a BD FACSAria™ III Sorter and then subjected to single cell RNA and immune repertoire sequencing. 1°: The first round of experiment; 2°: The second round of experiment. **b** Lymphocytes isolated from mouse were visualized by either tissue types (left) or cell subsets (right) with UMAP. **c** Proportional contribution of the three tissues (B, BM, and LN) to T/B clusters. The apparatus illustration was obtained from the official website of 10× Genomics (https://www.10xgenomics.com/instruments/chromium-x-series).

To identify and comprehensively characterize the T/B biphenotypic lymphocytes while minimizing the possibility of single-event artifacts, we conducted two rounds of single cell RNA sequencing (Fig. 1a). In the initial round of experiment, we isolated lymphocytes from mouse bone marrow and lymph node and performed single-cell RNA and immune repertoire sequencing. In a subsequent round of experiment, we expanded our scope to include lymphocytes from bone marrow and lymph nodes, as well as peripheral blood, thereby augmenting the diversity of our analytical dataset. Then, we conducted an integrated analysis merging the data derived from both sequencing rounds, incorporating information from all five samples. Cells that passed the quality control underwent unsupervised clustering and were displayed on a single UMAP space (Fig. 1b). Based on the transcription of marker genes (Supplementary Fig. 1), we identified clusters of T cells, B cells, neutrophils, erythroid cells, macrophages, NK cells, dendritic cells and hematopoietic stem cells (Fig. 1b). Intriguingly, in addition to these regular cell populations, we observed a subset of lymphocytes co-expressing both T- and B-cell lineage markers (Fig. 1b, Cluster 13), such as CD3d, CD4, CD8a, CD19, and Ms4a1 (Supplementary Fig. 1). These T/B biphenotypic cells were predominantly derived from blood and lymph node, with a smaller fraction originating from bone marrow (Fig. 1c).

### T/B biphenotypic lymphocytes identified in mouse coexpress TCR and BCR and may originate from pro/pre B precursors

**Fig. 2:**
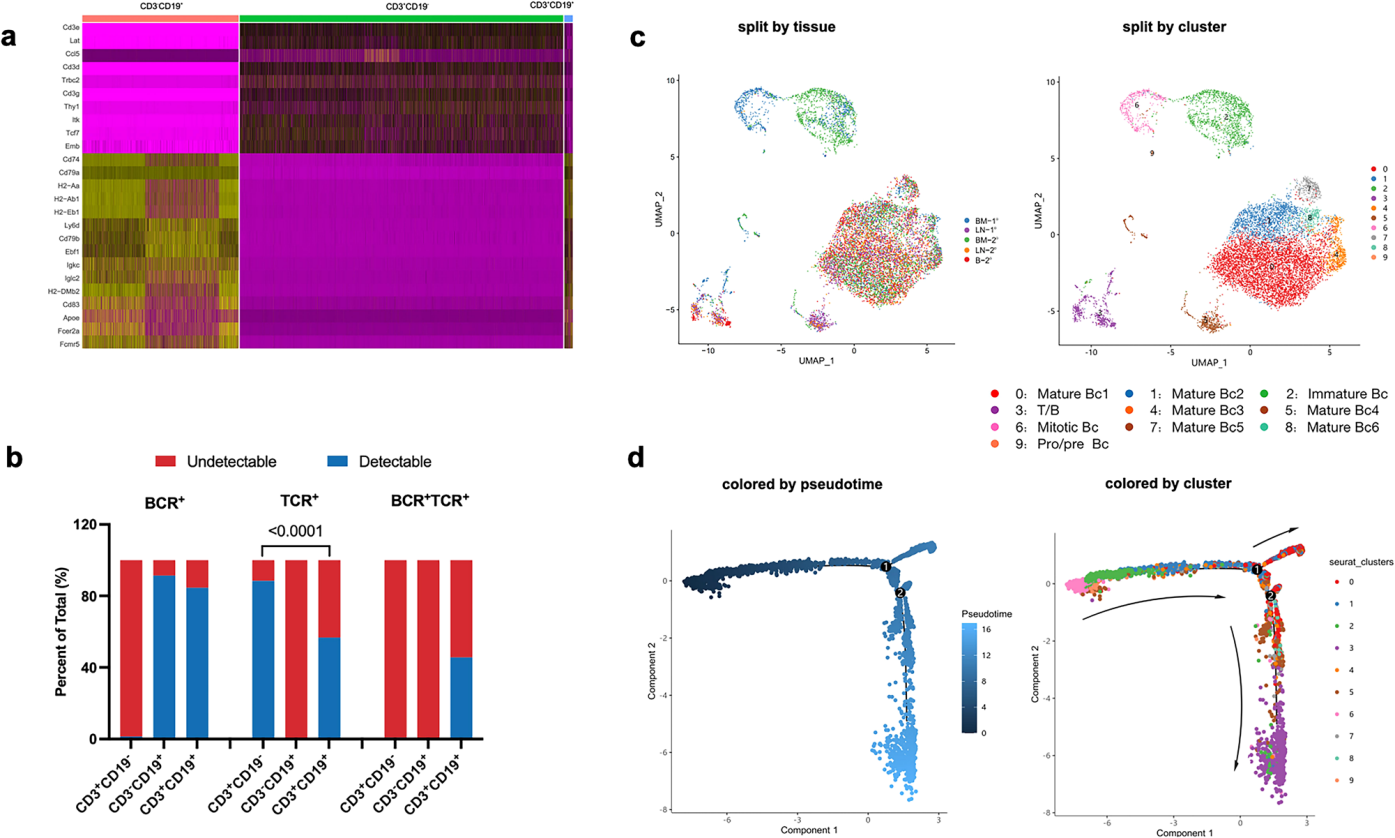
T/B biphenotypic lymphocytes identified in mouse coexpresses TCR and BCR and might originate from pro/pre B precursors. **a** The top 10 signature genes transcribed within each cluster. **b** The frequency of BCR and TCR contigs detected in CD3^+^CD19^-^, CD3^-^CD19^+^ and CD3^+^CD19^+^ cells. Statistical analyses were performed by the method of Chi-square test. **c** Cells with detectable BCR contigs were visualized by either tissue types (left) or cell subsets (right) with UMAP. **d** The cell development trajectories were analysised using Monocle 2, colored by pseudotime (left) or cluster (right).

To further characterize T/B biphenotypic lymphocytes, we used CD3 and CD19 as the primary lineage markers to discern conventional T cells (CD3^+^CD19^-^), conventional B cells (CD3^-^CD19^+^) and T/B biphenotypic cells (CD3^+^CD19^+^). The transcriptomic and immune repertoire profiles of the T/B biphenotypic cells were then compared with those of conventional T- and B-cells. Figure 2a illustrates the top 10 signature genes expressed within each cluster, revealing that the CD3^+^CD19^+^ cells possessed transcriptional attributes indicative of both T and B cell lineages. As expected, T-cell receptor (TCR) and B-cell receptor (BCR) repertoires analysis showed that most CD3^-^CD19^+^ B cells exhibited detectable contigs of BCR genes and most CD3^+^CD19^-^ T cells showed detectable contigs of TCR genes (Fig. 2b and Supplementary Data 1). Intriguingly, we observed that 46% of the CD3^+^CD19^+^ cells contained both BCR and TCR transcripts (Fig. 2b). The frequency of BCR contigs detected in CD3^+^CD19^+^ cells was comparable to that of CD3^-^CD19^+^ cells, while the frequency of TCR contigs detected in CD3^+^CD19^+^ cells was significantly lower than that in CD3^+^CD19^-^ cells (Fig. 2b). Similarly, clonotypes analysis revealed that 10 out of 310 BCR clones of CD3^+^CD19^+^TCR^+^BCR^+^ cells were shared with CD3^-^CD19^+^ cells, while only 2 out of 310 TCR clones overlapped with those of CD3^+^CD19^-^ cells (Supplementary Fig. 2a).

These findings indicated that CD3^+^CD19^+^ cells might more likely originate from B cell precursors. In order to validate this speculation, we selected cells with detectable BCR contigs and subjected them to a UMAP clustering analysis for the purpose of elucidating their developmental trajectory. This procedure yielded 10 distinct clusters (Fig. 2c and Supplementary Fig. 2b). Given that we did not know the exact starting point of the development of CD3^+^CD19^+^ cells, we firstly employed Monocle 2 for pseudotime analysis. The results revealed that the commencement of this trajectory was marked by early-stage B cells, including pro/pre B cells, mitotic B cells, and immature B cells (Fig. 2d). Mature B cells were evenly distributed in intermediate range of the trajectory. T/B cells traced its origin back to early-stage B cells and progressed through the developmental stage of mature B cells. To further validate and corroborate this finding, we set early developmental B cells, including mitotic B and pro/pre-B cells, as the starting point and conducted additional analysis using Monocle 3. The data consistently showed that the development of T/B biphenotypic cells progressed through a mature B cell subset (Supplementary Fig. 2c). Taken together, these results show the existence of T/B biphenotypic lymphocytes and imply that this cell subset might originate from pro/pre B precursors in mice.

### Flow cytometry assays confirmed the expression of key markers in the biphenotypic lymphocytes

**Fig. 3:**
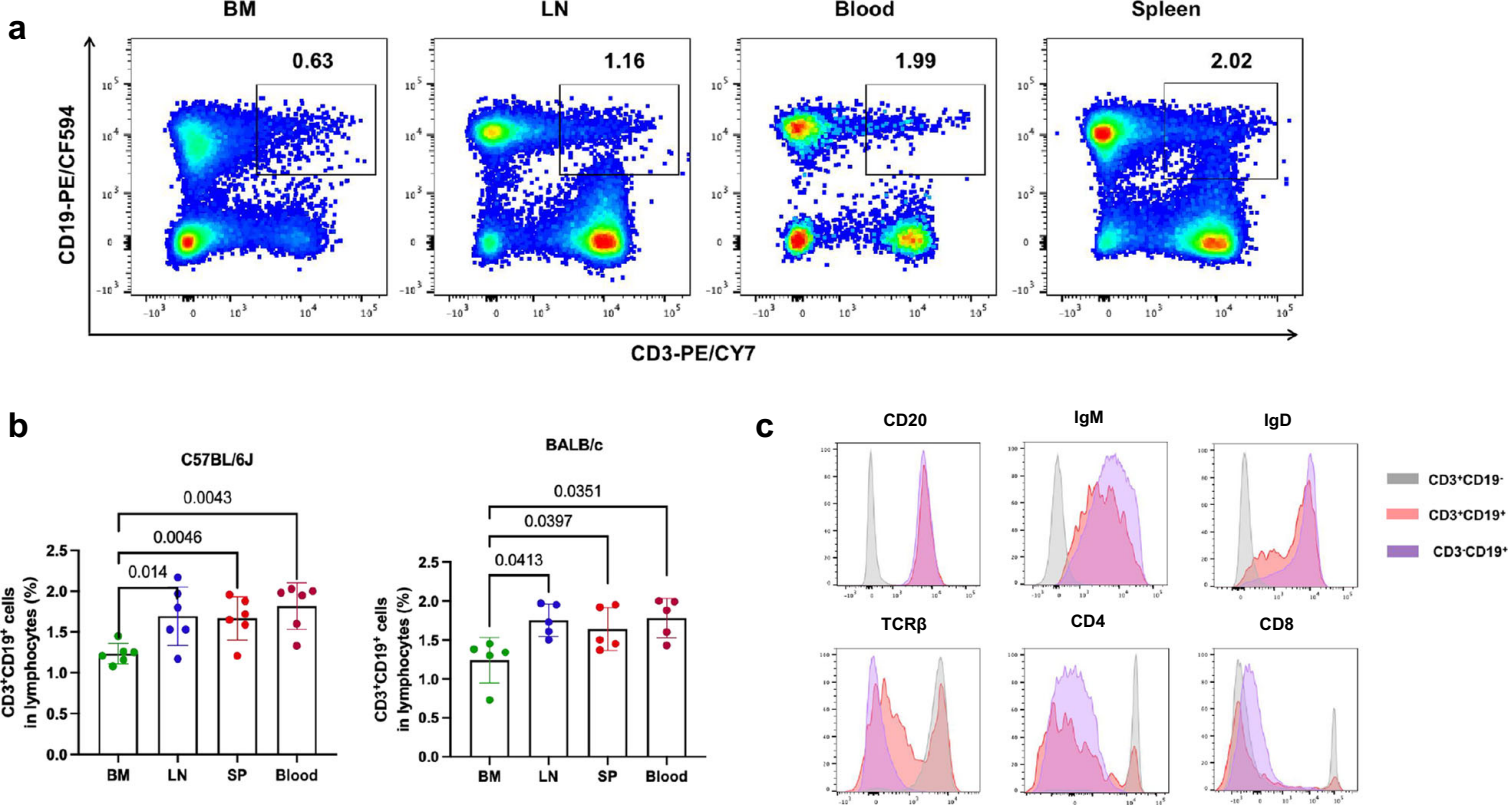
Flow cytometry assays confirmed the expression of key T- and B-cell markers in the biphenotypic lymphocytes. **a** Representative dot plots showed coexpression of CD3 and CD19 in mouse BM, LN, SP (spleen), and blood. Numbers indicate percentages in quadrants. **b** The percentages of CD3^+^CD19^+^ cells in the BM, LN, SP, and Blood were compared in C57BL/6 J and BALB/c mice, respectively. Data were shown as mean ± SD. All error bars represent SD. Statistical analyses were performed using the method of t-test. **c** The phenotype of CD3^+^CD19^+^ cells was characterized in parallel with CD3^+^CD19^-^ T cells and CD3^-^CD19^+^ B cells by flow cytometry analysis. One representative figure was shown for each surface marker.

Next, we verified the expression of T- and B-cell markers identified by scRNA-seq using flow cytometry assays. To eliminate the potential impact of long-term storage on expression of cell surface antigens^23^, all samples were freshly isolated, stained and analyzed in this study. Our data showed that CD3^+^CD19^+^ cells could be readily detected in mouse BM, LN, peripheral blood and spleen (Fig. 3a and Supplementary Fig. 3 for gating strategy). The frequencies of CD3^+^CD19^+^ cells in the LN (C57BL/6 J: 1.695 ± 0.3584; BALB/c: 1.752 ± 0.2067), spleen (C57BL/6 J: 1.668 ± 0.2642; BALB/c: 1.638 ± 0.2753) and peripheral blood (C57BL/6 J: 1.815 ± 0.2854; BALB/c: 1.780 ± 0.2537) were significantly higher than those in BM (C57BL/6 J: 1.235 ± 0.1244; BALB/c: 1.240 ± 0.2904) (Fig. 3b), which was in accordance with the scRNA-seq finding (Fig. 1c). The biphenotypic cell subset could be consistently detected when being stained with a panel of two anti-mouse CD3 and two CD19 mAb clones labeled with different fluorochromes (Supplementary Fig. 4a and 4b). Furthermore, the co-expression of CD3 and CD19 on individual cells was also visualized through immunofluorescence imaging (Supplementary Fig. 4c). We further examined the presence of other key T and B cell markers in CD3^+^CD19^+^ cells (Fig. 3c) and found that the expression of B cell markers (CD20, IgD, and IgM) was comparable with those of conventional B cells, while T-cell markers other than CD3 (TCR-β, CD4, and CD8) were less frequently expressed than those of conventional T cells.

A rare CD5^+^CD19^+^ lymphocyte subset was shown to simultaneously express TCR and BCR by a recent study^21^. To ascertain whether the CD3^+^CD19^+^ lymphocytes identified in our study and the previously reported CD5^+^CD19^+^ cells belong to the same subset, we followed the staining methodology outlined in the previous study and incorporated anti-mouse CD5 into our staining antibody panel (Supplementary Fig. 5). Our data showed that both CD3^+^CD19^+^ and CD5^+^CD19^+^ cells could be detected in mice, but these two populations only partially overlapped with each other. More specifically, 40% of the CD3^+^CD19^+^ cells expressed CD5 (CD3^+^CD19^+^CD5^+^) (40.05 ± 6.859, mean ± SD, *n* = 6). Lymphocytes coexpressing IgD and TCR-β were predominantly found within the CD3^+^CD19^+^CD5^+^ subset, while being conspicuously rare in the CD3^-^CD5^+^ CD19^+^ subset (Supplementary Fig. 5a) in mice.

### CD3^+^CD19^+^ cells displayed functional characteristics of both T and B cells

**Fig. 4:**
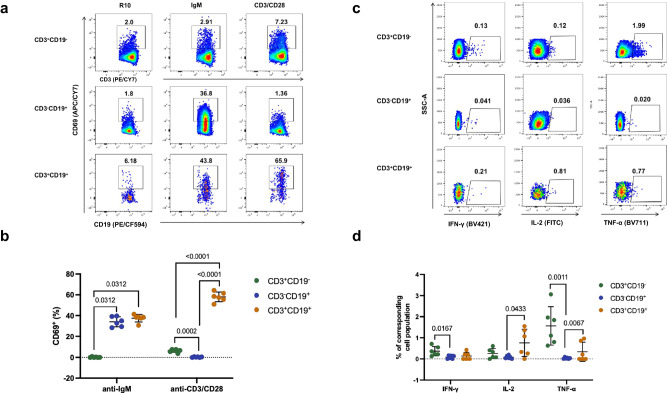
CD3^+^CD19^+^ cells in naive mice responded to stimuli of both anti-CD3/CD28 and anti-IgM. Splenocytes were collected from naïve C57BL/6 J mice and stimulated with anti-CD3/CD28, anti-IgM or R10. **a** Representative gating plots for measuring the expression of CD69. **b** Comparisons of the expression of CD69 in different cell subsets (mean ± SD, *n* = 6). **c** Representative plots for FACS gating of cytokine-secreting cells (IFN-γ, IL-2, or TFN-α). **d** Comparisons of the frequencies of IFN-γ, IL-2 and TFN-α secreting cells in different subsets (mean ± SD, *n* = 6). All error bars represent SD. Statistical analyses were performed using the method of t-test.

To characterize the functionality of the T/B biphenotypic lymphocytes, we first stimulated mouse splenocytes with either anti-CD3/CD28 or anti-IgM antibody and assessed the expression of the activation marker CD69. As being expected, anti-IgM stimulation upregulated CD69 expression in conventional (CD3^-^CD19^+^) B cells, but not in conventional (CD3^+^CD19^-^) T cells, whereas anti-CD3/CD28 stimulation upregulated CD69 in conventional T cells, but not in conventional B cells (Fig. 4a, b). Remarkably, the T/B biphenotypic (CD3^+^CD19^+^) cells responded to the stimuli of both anti-CD3/CD28 and anti-IgM (Fig. 4b). More intriguingly, we found that the anti-CD3/CD28 stimulation led to a more robust upregulation of CD69 in the T/B biphenotypic cells than in conventional T cells (Fig. 4b). In addition to monitoring the expression of CD69, we also measured the cytokine secretion upon anti-CD3/CD28 stimulation using the method of intracellular cytokine staining. Our results showed that both the conventional T cells and the T/B biphenotypic cells demonstrated their capacities to produce IFN-γ, IL-2, and TFN-α (Fig. 4c). The frequency of IL-2 secreting cells in the biphenotypic cells even tended to be higher than that in the conventional T cells (Fig. 4d).

**Fig. 5:**
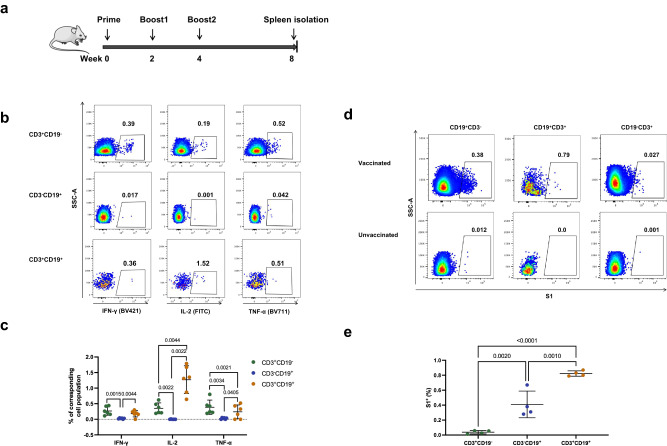
CD3^+^CD19^+^ cells in vaccinated mice responded to antigen-specific stimulation. **a** Female C57BL/6 J mice were immunized intramuscularly with a DNA vaccine encoding SARS-CoV-2 S1 protein for three times at an interval of 2 weeks. Four weeks post the third vaccination, the mice were euthanized and splenocytes were collected for assays of antigen-specific cellular immune responses. **b** Representative plots for FACS gating of S1 protein-specific cytokine-secreting cells (IFN-γ, IL-2, or TFN-α). **c** The frequencies of S1 protein specific IFN-γ, IL-2 and TFN-α secreting cells in different subsets (mean ± SD, *n* = 6). **d** Representative plots for FACS gating of S1 protein binding cells. **e** The frequencies of S1 specific B cells in different subsets (mean ± SD, *n* = 5). All error bars represent SD. Statistical analyses were performed using the method of t-test.

To investigate whether the T/B biphenotypic cells may participate specific responses towards foreign antigens, we immunized mice with a DNA vaccine encoding SARS-CoV-2 S1 protein and measured the S1 specific immune responses at 4 weeks post the 3^rd^ dose (Fig. 5a). In accordance with the results of pan-T cell stimulation, vaccination induced significantly higher frequencies of S1 specific cytokine secreting cells among the T/B biphenotypic subset than those among the conventional B subset (Fig. 5b, c). More specifically, the frequency of TFN-α secreting cells among the biphenotypic subset was significantly lower than that among conventional T cells, while the frequency of IL-2 producing cells was much higher in the biphenotypic subset than in the conventional T cell subset (Fig. 5c). Moreover, we also measured the frequencies of S1 specific B cells by staining with the purified S1 protein. The results showed that the average frequency of S1-specific B cells in the biphenotypic cells was approximately 2-fold of that in the conventional B cells (Fig. 5d, e). Collectively, these results suggested that the biphenotypic (CD3^+^CD19^+^) cells possessed functional characteristics of both T and B cells.

### Bifunctional CD3^+^CD19^+^ cells were also present in human peripheral blood

**Fig. 6:**
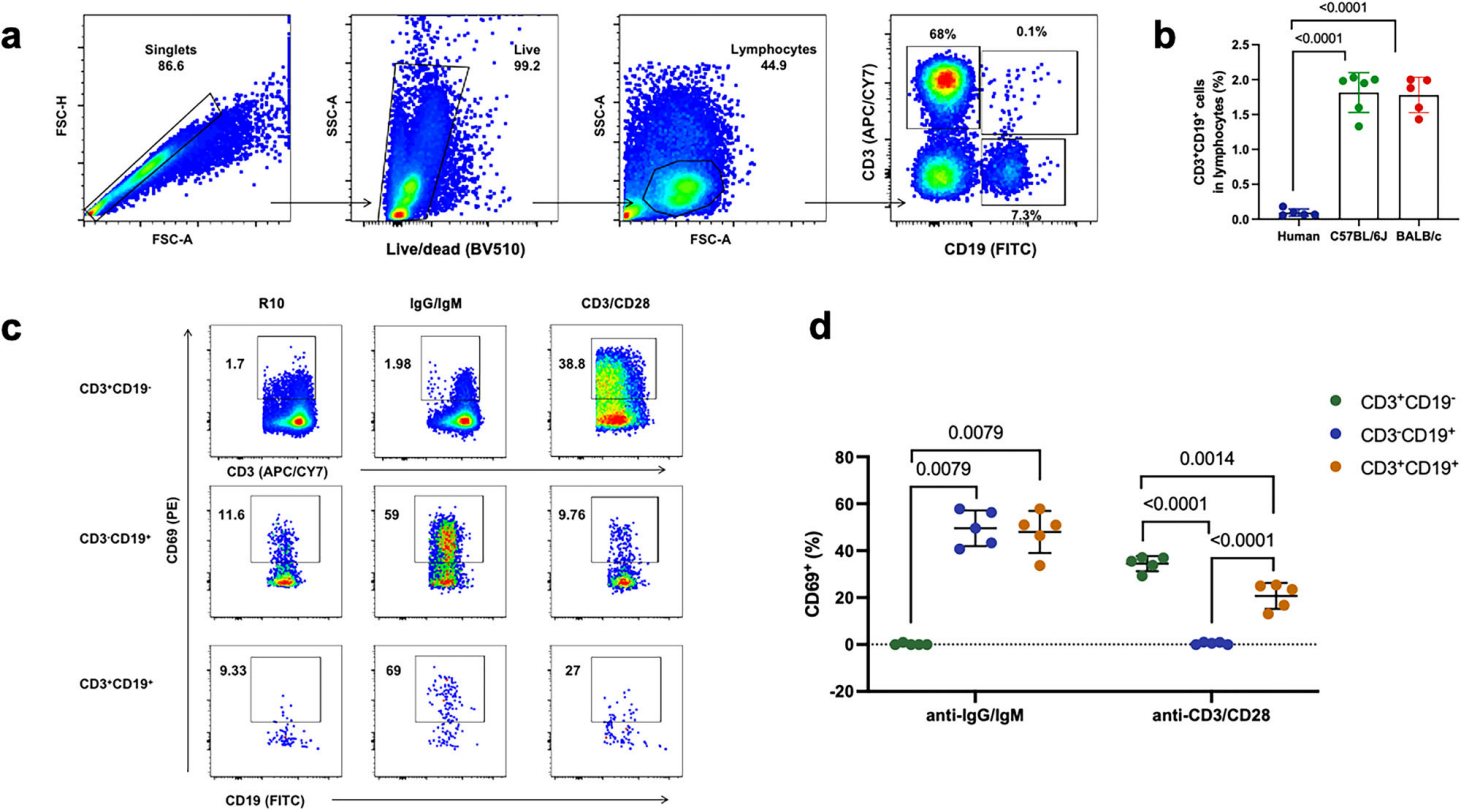
Functional CD3^+^CD19^+^ cells were also detected in humans. **a** The gating strategy for detecting CD3^+^CD19^+^ lymphocytes in human PBMCs. **b** The percentages of CD3^+^CD19^+^ cells in human and mouse peripheral blood lymphocytes were compared. **c** Representative plots showing the expression of CD69 after stimulating freshly isolated human PBMCs with anti-human CD3/CD28, anti-human IgG/IgM (H + L) or R10. **d** Comparisons of CD69 expression in different lymphocyte subsets (mean ± SD, *n* = 5). All error bars represent SD. Statistical analyses were performed using the method of t-test.

Flowcytometry analysis showed that CD3^+^CD19^+^ lymphocytes could also be detected in human peripheral blood, whereas the frequencies in lymphocytes (0.09 ± 0.05, *n* = 5) were lower than those of mice (Fig. 6a, b). We also characterized the functionality of these cells using the same method as being used for the analysis of mouse samples. Consistent with the findings in mice, our results showed that the T/B biphenotypic (CD3^+^CD19^+^) cells responded to the stimuli of both anti-human CD3/CD28 and anti-human IgG/IgM (H + L) (Fig. 6c, d).

## Discussion

T cell/B cell mixed phenotypic lymphocytes, such as CD3^+^CD20^+^^8,15^ and CD3^+^CD19^+^ cells^21,22^, have been identified by previous studies. However, other studies pointed out that the presence of T/B biphenotypic lymphocytes might be a bewildering phenomenon caused by technical issues^23,24^. In this study, we firstly proved that CD3^+^CD19^+^ lymphocytes could be steadily detected in both the central and peripheral lymphoid tissues of naive mice by the methods of single cell RNA-seq and flow cytometry. As all samples were freshly isolated and detected, we minimized the potential impact of storage on the phenotype of lymphocyte^23^. Our results showed that the biphenotypic lymphocytes endogenously expressed an array of T- and B-cell markers in addition to CD3 and CD19, including TCR-β, CD4, CD8, CD74, immunoglobin and so on, which differed from previously reported CD3^+^CD20^+^ lymphocytes that lacked CD19 and immunoglobulin molecules^10,14,17^. Moreover, the pseudo-time analysis of single-cell RNA-seq data showed that the majority of CD3^+^CD19^+^ subset might differentiate from precursors of pro/pre B cells, while the previously reported CD3^+^CD20^+^ cells were believed to be a population of CD3^+^ T cells co-expressing CD20^8,15^.

The B-lineage origin of T cell/B cell mixed phenotypic lymphocytes was also supported by both immune repertoire analyses and phenotypic characterization. CD3^+^CD19^+^ cells exhibited a relatively high frequency of overlapped BCR clones with conventional B cells. In contrast, they displayed a relatively frequency of overlapped TCR clones with conventional T cells. Besides, this argument was reinforced by the results that B cell markers such as CD20, IgD, and IgM were uniformly expressed in this cell subset, whereas T-cell markers, namely TCR-β, CD4, and CD8, were expressed less frequently compared to conventional T cells. A previous study showed that mature B cells could be turn into T cells via conditional Pax5 deletion^25^. Our research findings suggested a level of naturally occurred plasticity within the B cell lineage that cannot be attributed to Pax5-mediated reprogramming, because wild-type mice were used in this study and the transcription levels of Pax5 were comparable between the CD3^-^CD19^+^ and the CD3^+^CD19^+^ cells. This intriguing revelation underscores the complexity of immune cell development and the need for further investigations to elucidate the underlying mechanisms driving the emergence of these T/B biphenotypic cells.

Furthermore, our results demonstrated that the CD3^+^CD19^+^ biphenotypic cells in mice and humans could carry out both T- and B-cell functions. This subset of lymphocytes also participated antigen specific adaptive immune responses elicited by vaccination, as evidenced by the increased frequency of antigen-binding cells compared to conventional B cells and the similar level of antigen specific IFN-γ production with that of conventional T cells. Interestingly, the CD3^+^CD19^+^ subset produced a lower level of TNF-α, a pro-inflammatory cytokine^26^, but a higher level of IL-2, which is a growth factor essential for the proliferation and differentiation of T cells^27^. The unique functional characteristics indicated that the CD3^+^CD19^+^ subset may play a beneficial role in maintaining adaptive immunity. In addition, both our scRNA-seq and flow cytometry analyses revealed markedly higher frequencies of CD3^+^CD19^+^ cells in the lymph nodes and peripheral blood relative to the bone marrow, which suggested a plausible scenario wherein CD3^+^CD19^+^ cells generated within the bone marrow migrate to peripheral tissues, where they may respond actively to immune challenges. Further in-depth investigations are warranted to unravel the precise functional attributes and underlying regulatory mechanisms governing these intriguing cells in diverse anatomical contexts.

A subset of CD5^+^CD19^+^ lymphocytes expressing both B and T cell receptors, termed double expresser (DE) cells, was recently found in Type 1 Diabetes (T1D) patients^21^. However, it is still controversial whether this cell population is linked with the onset of T1D^28,29^. In consistence with previous reports, our study confirmed the presence of T/B biphenotypic cells, but the phenotype of the CD3^+^CD19^+^ lymphocytes identified in our study was not exactly the same with previously reported CD5^+^CD19^+^ DE cells. Although both CD3^+^CD19^+^ and CD5^+^CD19^+^ cells could be detected in mice, but these two populations only partially overlapped with each other. These findings collectively highlight the phenotypic and functional diversities of the T/B hybrid cells, which necessitates further research to thoroughly understand their complexities.

As aforementioned, a major limitation of this study is that the differentiation mechanism and the biological significance of the CD3^+^CD19^+^ biphenotypic lymphocytes were not fully clarified. Nevertheless, our study showed that these biphenotypic lymphocytes possessed both phenotypic and functional characteristics of conventional T and B cells, and implied that this lymphocyte subset might play a role in regulating adaptive immunity. Further investigations are warranted to elucidate the precise mechanisms governing the development and the functionality of these biphenotypic lymphocytes, as well as their potential contribution to immune responses in different physiological and pathological contexts.

## Methods

### Ethics statement

We have complied with all relevant ethical regulations for animal use. All ethical regulations relevant to human research participants were followed. Informed consent was obtained for the collection and use of human peripheral blood samples. Experiments using mice and human peripheral blood samples were approved by the Research Ethics Review Committee of the Shanghai Public Health Clinical Center Affiliated to Fudan University.

### Single cell RNA sequencing and T/B cells immune repertoire profiling

To prepare samples for scRNA-seq analysis, bone marrow, lymph node and peripheral blood cells isolated from C57BL/6 J female mice were stained with Live/Dead dye (Fixable Viability Stain 510, cat# 564406, BD Pharmingen) and lymphocytes were isolated using a BD FACSAria™ III Sorter (Fig. 1a). Cells were manually counted by Trypan blue (Thermo, T10282) and AO-PI (LUNA, D23001) after each centrifugation and resuspension. Single cells were processed using Chromium Controller (10x Genomics) according to the manufacturer’s protocol. By using Chromium Next GEM Single Cell 5’ Kit v2 (10x Genomics, 1000263) and Chromium Next GEM Chip K Single Cell Kit (10x Genomics, 1000287), we performed single cell TCR/BCR-seq and 5’ gene expression profiling. The cell suspension was loaded onto the Chromium single cell controller (10x Genomics) to generate single-cell gel beads in the emulsion according to the manufacturer’s protocol. Captured cells were lysed and the released RNA were barcoded through reverse transcription in individual GEMs. Cell-barcoded 5’ gene expression libraries and V(D)J enriched TCR/BCR libraries were sequenced on an Illumina NovaSeq6000 system by Shanghai Biochip Co., Ltd., Shanghai, China.

### scRNA-seq data analysis

Sequencing reads from gene expression and V(D)J libraries were aligned to mm10 mouse reference genome using cellranger 7.0.0 with “multi” mode. The generated count matrices and V(D)J contig annotations were used for downstream analysis performed with Seurat (v4.1.1) and Scirpy (v0.11.2). All samples were aggregated into a single SeuratObject. Cells with gene number (<300 & >97.5% quantile) or high mitochondrial transcript ratio (>25%), and genes expressed in less than 3 cells were excluded. After removing unwanted cells from the dataset, all samples were combined with function “merge”. Next, we employed a global-scaling normalization method “LogNormalize” to normalize the feature expression measurements (UMI counts) for each cell by the total expression, then data integration was performed by canonical correlation analysis according to shared sources of variation across multiple datasets using SelectIntegrationFeatures, FindIntegrationAnchors and IntegrateData functions. Highly variable genes (top 3000) were extracted to perform the principal component analysis (PCA) and top 30 of significant principle components were used for cluster analysis. Clusters were visualized using the Uniform Manifold Approximation and Projection (UMAP). Marker genes for each cluster and subgroup were identified by contrasting gene expression of cells from certain cluster or subgroup to that of others using the Seurat FindMarkers function.

### BCR/TCR analysis

For TCR and BCR data, we followed the typical Scirpy analysis workflow. Scirpy implements a network-based approach for clonotype definition based on identical or similar CDR3 amino acid sequences, which enables us to identify cells that might recognize the same antigen. Briefly, the distances between CDR3 nucleotide(nt) or amino acid (aa) sequences were measured using function scirpy.pp.ir_dist with parameters “metric = ‘ identity’ sequence = ‘nt’” and “metric = ‘alignment’ sequence = ‘aa’” respectively, either based on sequence identity or similarity. In this study, clonotypes were defined based on nt-sequence identity using scirpy.tl.define_clonotypes with “dual_ir = ‘primary_only’ same_v_gene = True”.

### Pseudotime analysis of scRNA-seq

Pseudotime trajectories for selected subclusters were constructed using Monocle 2 and Monocle 3 (v1.3.4). For monocle 2, the raw counts for cells in the intended subclusters were extracted and filtered top 2000 high variable Genes by Seurat v4.0.3, and then normalized by the estimateSizeFactors and estimateDispersions functions with monocle’s default parameters. Genes with an average expression >0.1 and detected in more than 50 cells were retained for further analysis. Variable genes were determined by the differentialGeneTest function with a model against the subcluster identities. The variable genes with the lowest adjusted qval < 0.0001 were used to order the cells. The orders were determined by the orderCells function and the trajectory was constructed by the reduceDimension function with default parameters.

For monocle 3, After constructing CellDataSet object using raw counts of selected cells, data were normalized and pre-processed to remove any batch effects using preprocess_cds and align_cds. The UMAP layout generated by Seurat was also imported into CDS object. Functions learn_graph and order_cells were employed to build trajectories and measure the pseudotime of each cell, subcluster 6 and 9 was selected as root nodes in this step. There are two approaches for differential analysis in Monocle3. Here, we identified the genes that change as cells progress along the pseudotime by graph-autocorrelation analysis using graph_test with neighbor_graph = “principal_graph” parameter, then genes with q value < 0.001 were selected. To find modules of co-regulated genes, we call find_gene_modules which groups genes into modules using Louvain algorithm. All trajectory graphs were visualized using plot_cells or plot_genes_in_pseudotime.

### Mouse vaccination

6 to 8-week-old female C57BL/6 J mice were housed under specific-pathogen free environment and immunized intramuscularly with a DNA vaccine encoding SARS-CoV-2 S1 protein (50 μg/mouse) for three times at an interval of 2 weeks. Four weeks post the third vaccination, the mice were euthanized and splenocytes were collected for assays of antigen-specific cellular immune responses.

### Cell stimulation

Freshly isolated splenocytes were plated into round-bottom 96-well plates (2×10^6^ cells per well) and incubated with R10 (RPMI1640 with 10% FBS), R10 containing synthesized peptides encompassing the full length of S1 protein (0.66 μg/ml for each peptide) (Synthesized by Gill Biochemistry Co., Ltd., Shanghai, China), R10 containing anti-CD3 (1 μg/mL) (cat# 16-0032-85, Invitrogen) and anti-CD28 (3 μg/mL) (cat# 16-0281-85, Invitrogen) or R10 containing F(ab’)2-Goat anti-Mouse IgM (10 μg/mL) (cat# 115-006-020, Jackson) for 7 h at 37 °C and 5% CO_2_. For staining of intracellular cytokine, brefeldin A was added to each well at a final concentration of 1 μg/ml at 1 h after the beginning of stimulation.

### Flow cytometric analysis

Freshly isolated cells were washed with phosphate-buffered saline (PBS) and stained sequentially with Live/Dead dye (Zombie Aqua™ Fixable Viability Kit, cat# 564406, Biolegend) for 15 min at room temperature (RT), Fc-receptor blockade (anti-mouse CD16/32 antibody, cat# 156604, Biolegend) for 10 min on ice and surface markers (PE/Cyanine7-labeled anti-mouse CD3, cat# 100220, BioLegend; APC-labeled anti-mouse CD3, cat# 100311, BioLegend; PE/Dazzle™ 594-labeled anti-mouse CD19, cat# 115554, BioLegend; FITC-labeled anti-mouse CD19, cat# 152403, BioLegend; APC-labeled anti-mouse CD5, cat# 100625, BioLegend; APC-labeled anti-mouse CD4, cat# 100412, BioLegend; PE- labeled anti-mouse CD8, cat# 100708, BioLegend; APC-labeled anti-mouse CD20, cat# 150412, BioLegend; Brilliant Violet 421-labeled anti-mouse IgM, cat# 406518, BioLegend; Brilliant Violet 785-labeled anti-mouse TCR-β, cat# 109249, BioLegend; APC/Cyanine7-labeled anti-mouse CD69, cat#104526, BioLegend) for 30 min at 4 °C, respectively. For staining of intracellular markers, stimulated cells were fixed and permeabilized using the BD Cytofix/Cytoperm kit (cat# 554714, BD Bioscience) according to the manual after surface staining, then stained with BV421-labeled anti-mouse IFN-γ (cat# 505830, BioLegend), FITC-labeled anti-mouse IL-2 (cat# 503806, BioLegend) and BV711-labeled anti-mouse TNF-α (cat# 506349, BioLegend) for 30 min at 4 °C. Stained samples were analyzed using a BD LSRFortessa flow cytometer and data were analyzed with the FlowJo software version 10 (TreeStar Inc., Ashland, OR, USA).

### Immunofluorescence

Lymph node cells isolated from C57BL/6 J female mice were stained with Alexa Fluor® 594 anti-mouse CD19 Antibody (cat# 115552, BioLegend) and Alexa Fluor® 488 anti-mouse CD3 Antibody (cat# 100210, BioLegend) for 30 min at 4 °C. Following the staining process, the cell suspension was placed into glass bottomed dishes. Once the cells settled naturally to the dish’s bottom surface, images were captured utilizing the Leica TCS SP5 II laser confocal microscopy system.

### FACS analysis of S1 specific B cells in mice

Splenocytes isolated from vaccinated mice were washed and stained sequentially with Live/Dead dye, biotinylated S1 protein (Cat#40591-V08H-B, Sino Biological, China) and surface markers (PE/Cyanine7-labeled anti-mouse CD3, cat# 100220, BioLegend; PE/Dazzle™ 594-labeled anti-mouse CD19, cat# 115554, BioLegend and Streptavidin-FITC, cat# 405201, BioLegend) respectively. After washing, the stained cells were resuspended in 200 µl 1× PBS and analyzed using a BD LSRFortessa™ Flow Cytometer.

### Collection and detection of human peripheral blood samples

Healthy adult peripheral blood was collected by venipuncture into ethylenediaminetetraacetic acid (EDTA) tubes and diluted with an equal volume of PBS. Subsequently, the diluted blood was carefully layered onto a sterile centrifuge tube containing a Ficoll density gradient and centrifuged at 400 g for 30 min. The resulting peripheral blood mononuclear cells (PBMCs) were collected and plated into round-bottom 96-well plates (2×10^6^ cells/well). Then, the PBMCs were incubated with R10, R10 containing anti-human CD3 (1 μg/mL) (cat# 16-0037-81, Invitrogen) and anti-human CD28 (3 μg/mL) (cat# 16-0289-81, Invitrogen) or R10 containing F(ab’)2-Goat anti-human IgG, IgM (H + L) (10 μg/mL) (cat# 16-5099-85, Invitrogen) for 4 h at 37 °C and 5% CO_2_. Cells were washed and stained sequentially with Live/Dead dye for 15 min at room temperature (RT) and surface markers (APC/Cyanine7-labeled anti-human CD3, cat#300318, BioLegend; FITC-labeled anti-human CD19, cat#302206, BioLegend; PE-labeled anti-human CD69, cat#3109056, BioLegend) for 30 min at 4 °C, respectively.

### Statistics and reproducibility

Statistical analysis, except for the single-cell RNA-seq-data, were performed using GraphPad Prism 9 (GraphPad Software, Inc., La Jolla, CA, USA). The number (n) of animals per group was indicated in the figure legends. Data were first analyzed by the Kolmogorov-Smirnov test to assess normal distribution. Comparisons between two groups were conducted by the method of t-test. *P* *<* *0.05* was considered as statistically significant.

### Reporting summary

Further information on research design is available in the Nature Portfolio Reporting Summary linked to this article.

### Supplementary information

Supplementary Information

Description of Additional Supplementary Files

Supplementary Data 1

Supplementary Data 2

Reporting Summary

## Data Availability

Data supporting the findings of this work are available within the paper and in the Supplementary files. The Sc-RNAseq data generated in this study is available in the Gene Expression Omnibus database (GSE249618). The source data behind the graphs in the manuscript is included in the Supplementary Data 2.
