# Supplementary material for: A biphenotypic lymphocyte subset displays both T- and B-cell functionalities
Source: Commun Biol. 2024 Jan 5;7:28. doi: 10.1038/s42003-023-05719-9 (PMC10770049; doi:10.1038/s42003-023-05719-9)
Supplement: Supplementary file 1 — Supplementary Information [file 42003_2023_5719_MOESM1_ESM.pdf]

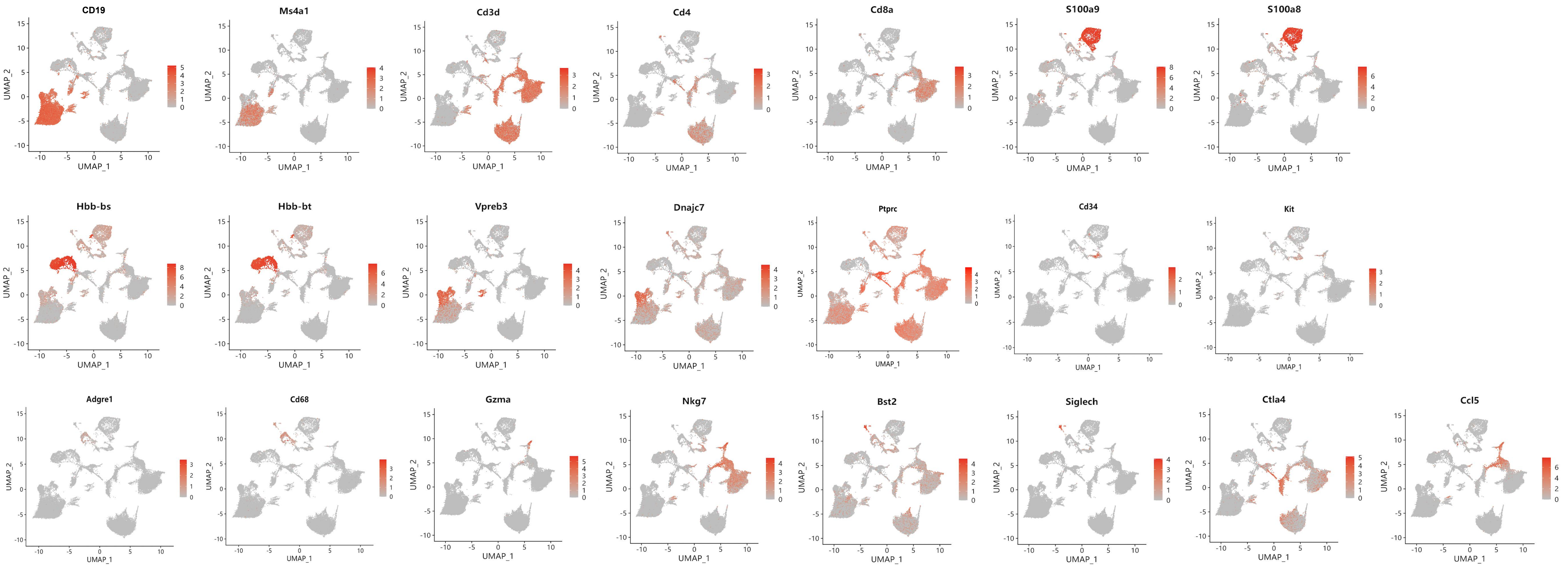

Supplementary Figure 1 The transcription of key lineage marker genes manifested by single cell RNA-seq. Visualization of the transcription of key lineage marker genes with UMAP. Mature B cells (CD19, Ms4a1), T cells (CD3d, CD4, CD8a), neutrophils (S100a8, S100a9), erythroid cells (Hbb-bs, Hbb-bt), immature B cells (Vpreb3, Dnajc7), lymphocytes (ptprc), hematopoietic stem cells, HSC (CD34, kit), macrophages (adgre1, CD68), NK cells (Gzma, Nkg7), dendritic cells (Bst2, Siglech) and T/B cells (CD3d, CD79a).

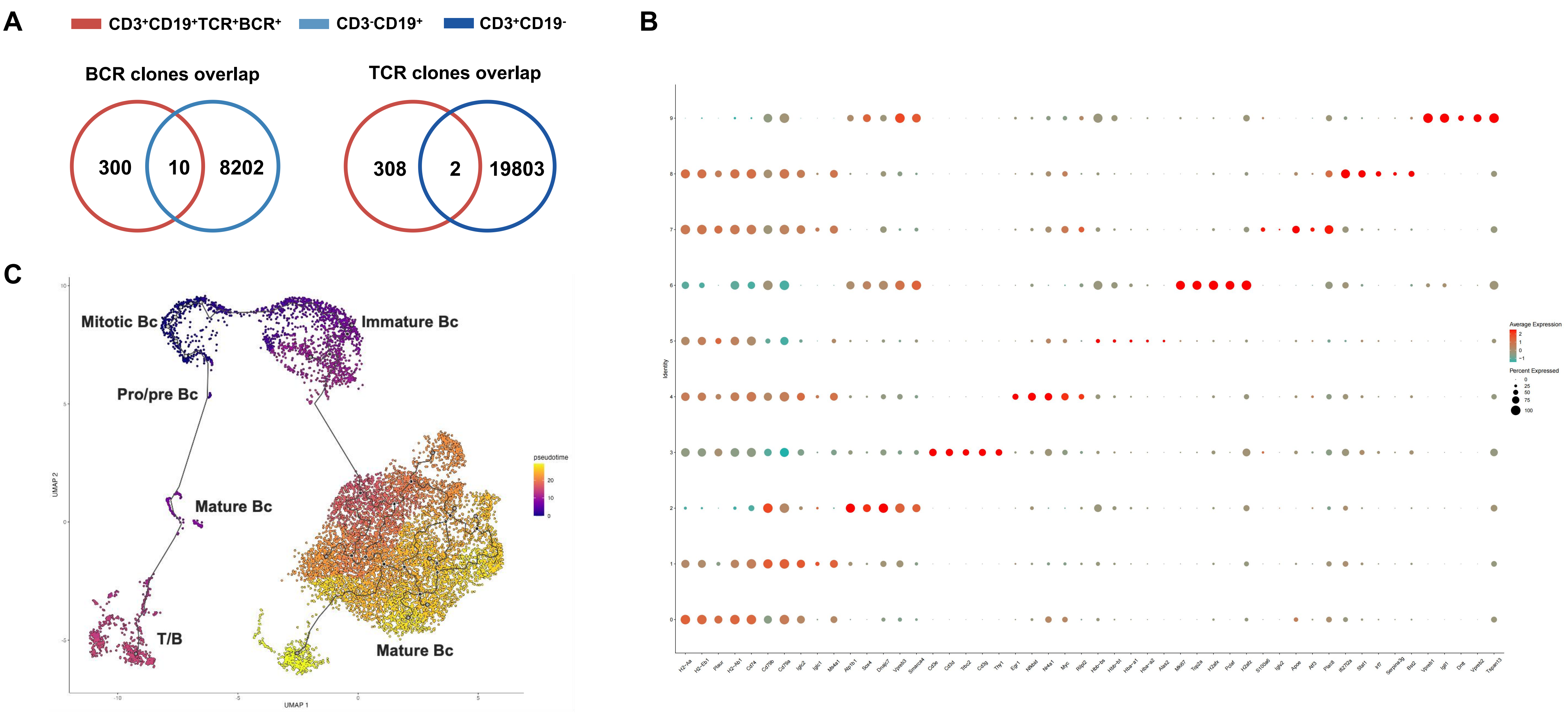

Supplementary Figure 2 T/B biphenotypic lymphocytes identified in mouse may originate from pro/pre B precursors. (**A**) Venn diagrams representing the number of detected BCR (or TCR) clones shared between CD3^{-}CD19^{+} cells (or CD3^{+}CD19^{-}) and CD3^{+}CD19^{+}TCR^{+}BCR^{+} cells. (**B**) Dot-plot representation of the gene expression marker genes for the identified cell types. Dot size encodes percentage of cells expressing the gene, color encodes the average per cell gene expression level. (**C**) UMAP visualization of the B cell trajectory colored by pseudotime as computed in Monocle 3. Corresponds to Figure 2C.

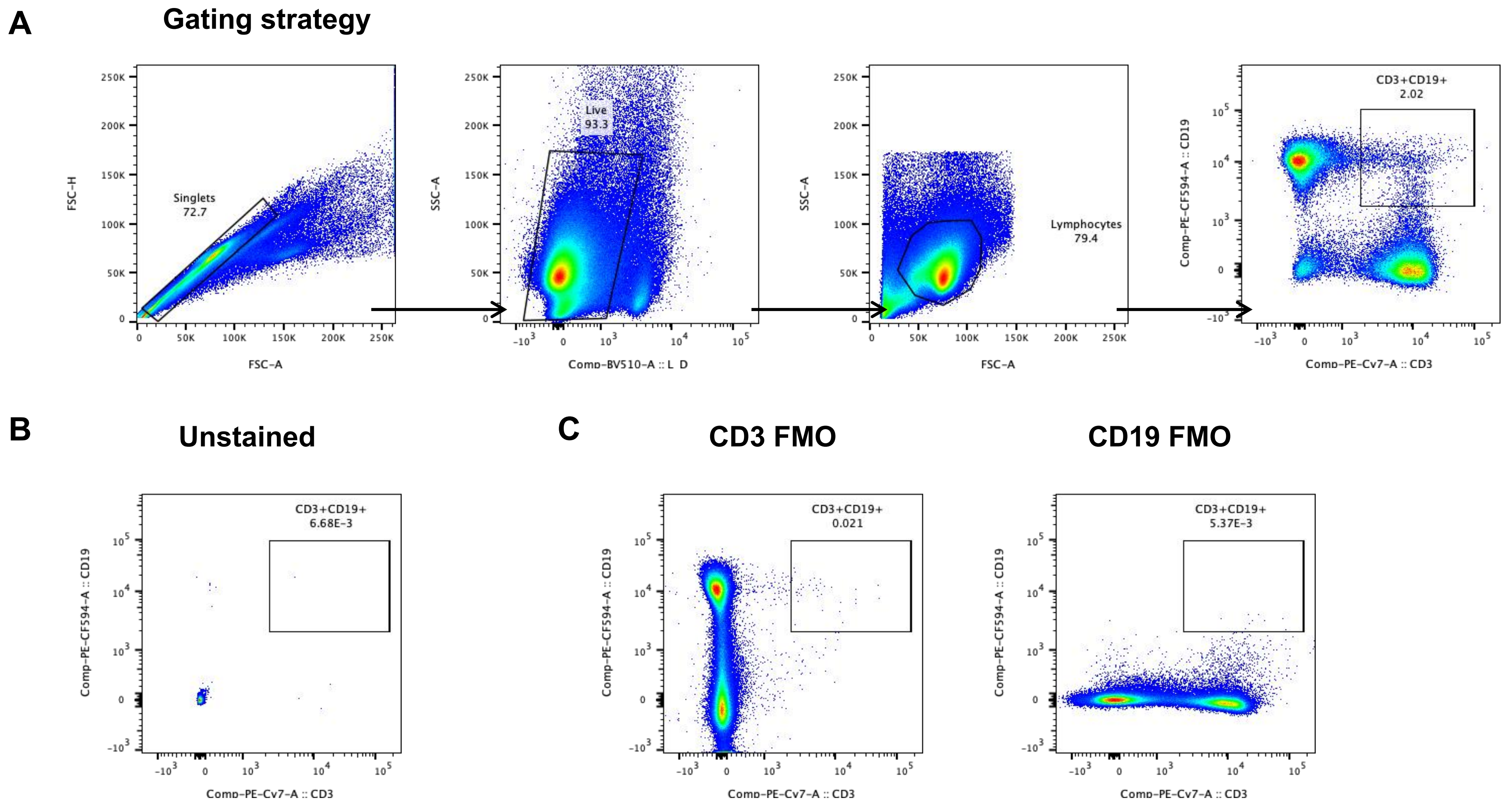

Supplementary Figure 3 Identify CD3⁺CD19⁺ cells by flow cytometry. (**A**) Flow cytometry gating strategy. (**B**) No CD3⁺CD19⁺ cells were detected in unstained samples. (**C**) FMO analyses showed no nonspecific signals for CD3 and CD19.

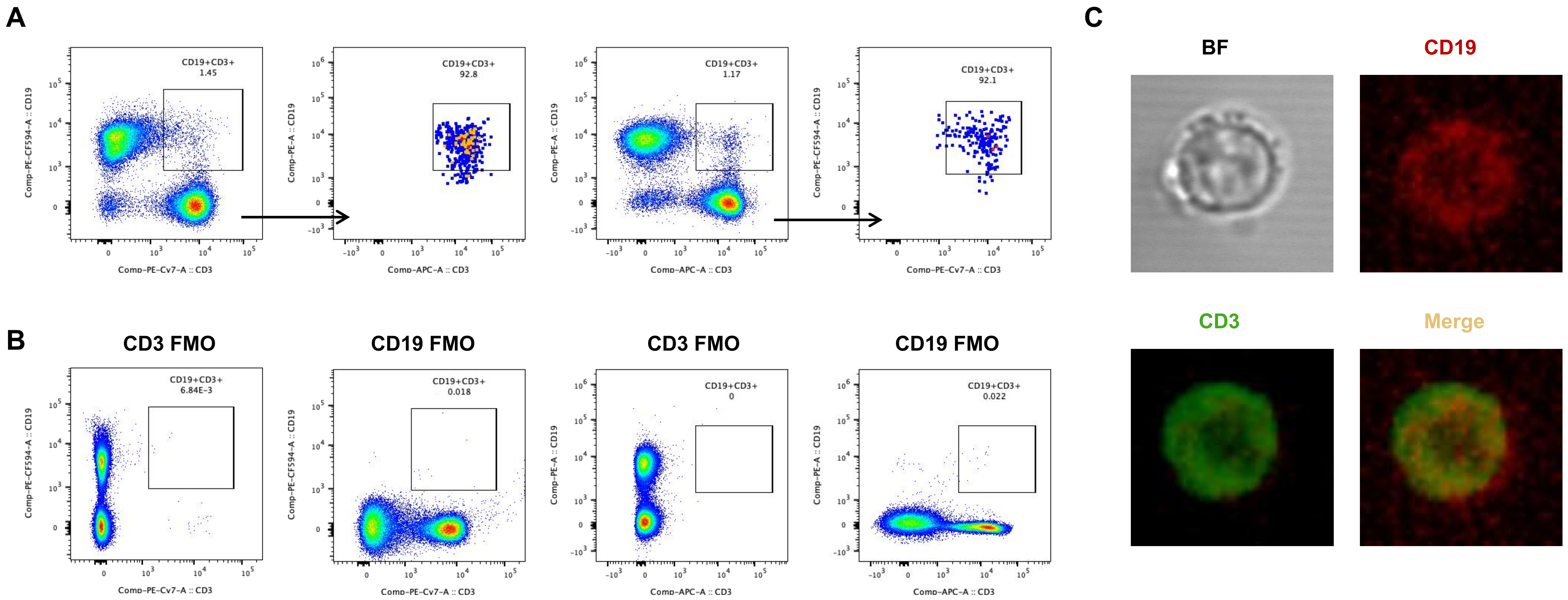

Supplementary Figure 4 Verification of CD3⁺CD19⁺ cells using different specificity controls. (**A**) Distinct clones of anti-mouse CD3 and CD19 mAbs labeled with different fluorochromes were used to confirm the accuracy of the flow cytometry findings. Flow cytometry gating strategy was consistent with Supplementary Figure 3A. (**B**) FMO analyses showed no nonspecific signals for CD3 and CD19. (**C**) Representative confocal microscopy images showed coexpression of CD3 and CD19 in mouse lymphocytes isolated from LN (n= 1 sample); scale bar, 7.5 μm. BF, bright field.

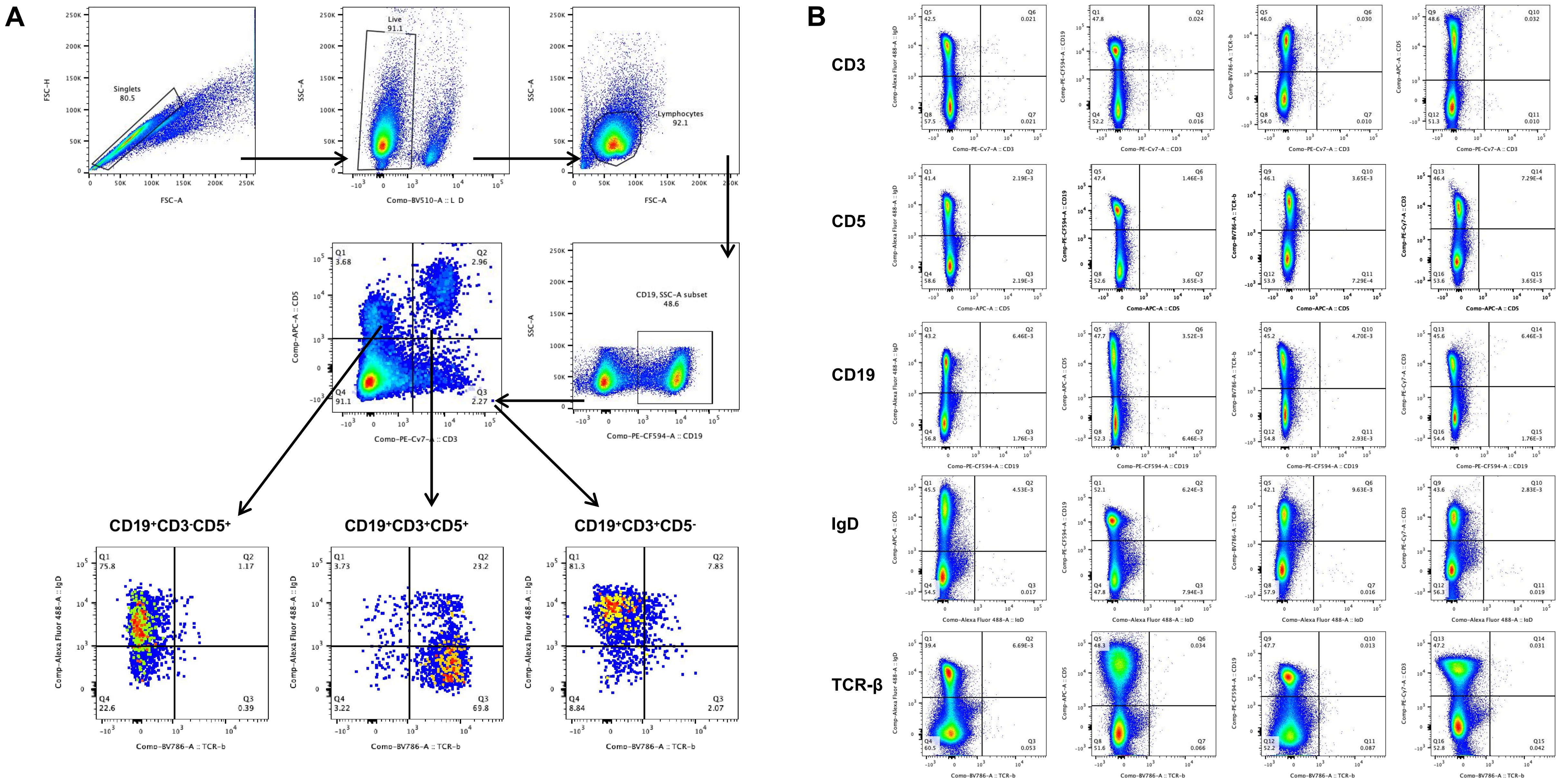

Supplementary Figure 5 CD3⁺CD19⁺ cells were not corresponded to CD5⁺CD19⁺ cells. (**A**) Flow cytometry gating strategy. (**B**) FMO analyses show no nonspecific signals for CD3, CD5, CD19, IgD, and TCR-β.
