# Supplementary material for: A biphenotypic lymphocyte subset displays both T- and B-cell functionalities
Source: Commun Biol. 2024 Jan 5;7:28. doi: 10.1038/s42003-023-05719-9 (PMC10770049; doi:10.1038/s42003-023-05719-9)
Supplement: Supplementary file 2 — Description of Additional Supplementary Files [file 42003_2023_5719_MOESM2_ESM.pdf]

Description of Additional Supplementary Files

File name: Supplementary Data 1
Description: TCR and BCR repertoires analysis.

File name: Supplementary Data 2
Description: The source data behind the graphs in the paper.
